# Advanced deep learning enables prediction of allogeneic stem cell mobilization success

**DOI:** 10.1038/s41409-026-02811-6

**Published:** 2026-03-17

**Authors:** Asif Adil, Jingyu Xiang, Nicola Piccirillo, Hillary G. Harris, Simona Sica, John F. DiPersio, Stephanie N. Hurwitz

**Affiliations:** 1 Department of Pathology and Laboratory Medicine, Indiana University, Indianapolis, IN USA; 2 Melvin and Bren Simon Comprehensive Cancer Center, Indianapolis, IN USA; 3 Division of Oncology, Department of Medicine, Washington University School of Medicine, St. Louis, MO USA; 4 Dipartimento di Scienze di Laboratorio ed Ematologiche, Fondazione Policlinico Universitario “A. Gemelli” IRCCS, Rome, Italy; 5 Sezione di Ematologia, Dipartimento di Scienze Radiologiche ed Ematologiche, Università Cattolica del Sacro Cuore, Rome, Italy

**Keywords:** Risk factors, Translational research, Prognosis

## Introduction

Efficient mobilization of donor hematopoietic stem and progenitor cells (HSPCs) to peripheral blood (PB) is essential to the success of HSPC transplantation [1]. Although granulocyte colony-stimulating factor (G-CSF) is widely used to induce mobilization of HSPCs, healthy donors exhibit marked inter-individual variability in CD34⁺ cell yield [2–4]. Across institutions, variability may be in part due to differences in mobilization regimens, dosing, and apheresis procedures. Inadequate mobilization can compromise cell collection, and has consequences, including delayed engraftment, graft failure, and relapse. Donors may also face complications of prolonged G-CSF treatment and multiple apheresis sessions [5]. However, there remains no reliable method to identify these individuals prior to apheresis collection [6].

Prior efforts to predict mobilization outcome have largely been confined to single-time-point analyses with limited accuracy [2–4, 7]. Pre-G-CSF models, while useful for early risk stratification, may lack predictive resolution in borderline donors. Conversely, post-G-CSF models capture real-time biological response but offer only reactive interventions. A combined modeling strategy that leverages both pre- and post-G-CSF data offers the dual advantage of early prediction and refined risk assessment once G-CSF has begun.

Here, we utilize baseline and post-mobilization laboratory values to improve accuracy of single-timepoint prediction models. We demonstrate the performance of advanced deep learning models that can flexibly weigh routine laboratory data from both baseline and post-GCSF timepoints to accurately predict poor mobilizers and support personalized decision-making throughout the mobilization timeline.

## Methods

The study was approved by the Indiana University (IU) Institutional Review Board (IRB). Demographic data and post-mobilization routine laboratory parameters were evaluated in allogeneic HSPC donors from 2019–2024 at IU (*n* = 203). Donors were mobilized with G-CSF; a minor subset of patients additionally received plerixafor. Post-mobilization data from healthy donors (*n* = 158) at the Università Cattolica del Sacro Cuore in Rome (CU) and pre-mobilization data from Washington University (WU) healthy donors (*n* = 799) were also included [2, 3]. To validate generalizability of our framework, we utilized an international donor cohort (*n* = 19,207) from the Center for International Blood and Marrow Transplant Research (CIBMTR) [8]. A cutoff of ≥40 CD34^+^ cells/μL defined “good mobilizers,” consistent with that used previously [2]. See Supplemental Methods for additional details.

## Results

**Fig. 1:**
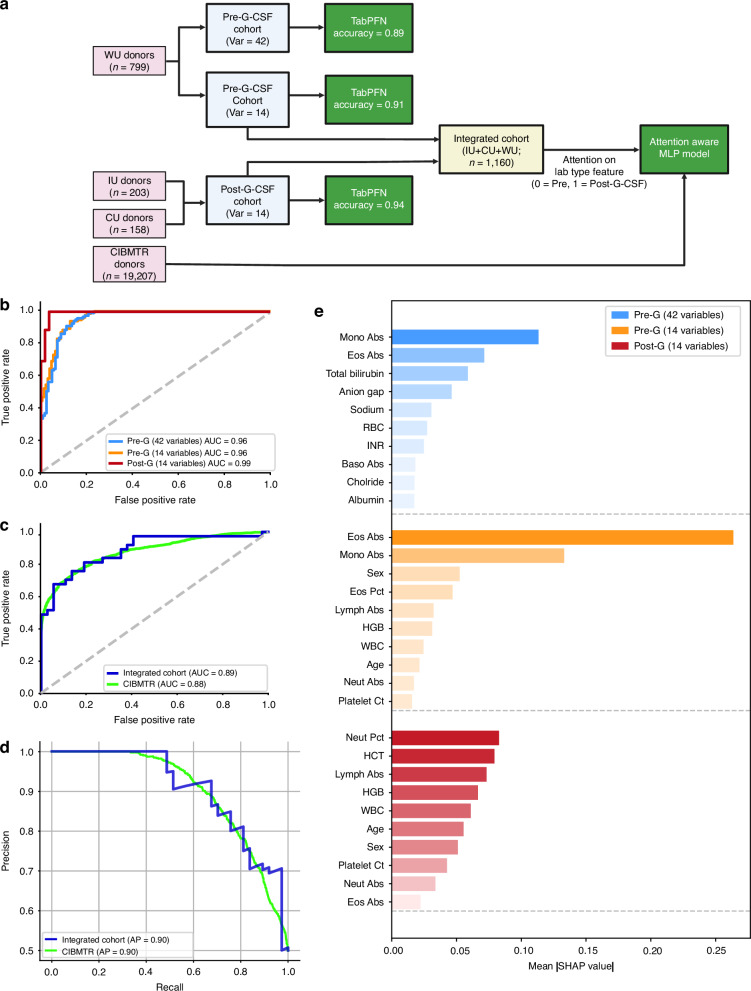
Deep-learning framework for predicting stem cell mobilization outcome from routine laboratory data. **a** Overview of model development and integration across cohorts. Washington University, WU; Indiana University, IU; Università Cattolica del Sacro Cuore in Rome, CU; Center for International Blood and Marrow Transplant Research, CIBMTR. **b** Receiver operating characteristic (ROC) curves for the held-out and independent test sets of TabPFN models. Dashed line indicates random classifier performance. **c** ROC curves for the attention-aware held-out test sets of the integrated and external CIBMTR cohorts. **d** Precision-recall curve illustrating attention-aware model performance across classification thresholds, maintaining a precision >0.80 across most recall values. **e** Global feature importance ranked by mean absolute SHAP values for the TabPFN models. Pre-G, Pre-G-CSF; Post-G, Post-G-CSF; Var, variables.

Using all four donor cohorts, we developed and evaluated predictive models for mobilization outcomes, using a structured analytical pipeline (Fig. 1a, S1).

Based on a calculated cutoff of ≥ 40 CD34⁺/μL, approximately 20% of donors were classified as poor mobilizers (Fig. S2a–c). Female and older donors were enriched among poor mobilizers (Fig. S2d, e), although sex alone was not an independent predictor. Notably, poorly mobilized male donors were older than male donors who mobilized successfully (*p* < 0.0001) (Fig. S2f).

To evaluate pre- and post-mobilization donors separately, we applied transformer-based probabilistic (TabPFN) models (Fig. 1b, S3). In the pre-G-CSF cohort (*n* = 799), TabPFN trained on all 42 baseline variables (Table S1) achieved an accuracy of 89% and area under the receiving operator curve (AUC) of 0.96. Restriction of variables to just demographic and CBC features (14 variables; Table S1) retained similar discrimination (accuracy = 91%, AUC = 0.96). Using the same 14 variables, TabPFN prediction in the post-GCSF cohort (*n* = 361) yielded an accuracy of 94% (AUC = 0.99).

To leverage the full integrated cohort of 1160 donors, we trained an attention-aware deep learning model that incorporates data from both timepoints with an explicit lab-type indicator (0 = pre-G-CSF, 1 = post-G-CSF) to indicate context (Fig. S3). This model achieved an accuracy of 81% with an AUC of 0.89 (Fig. 1c, S3). To further evaluate the generalizability and clinical relevance of the framework, we applied it to a large CIBMTR dataset of healthy donors (*n* = 19,207) with pre- and post-G-CSF laboratory values. Despite using only nine reported features (Table S1), the model achieved an accuracy of 79% (AUC = 0.88) (Fig. 1c, S3). Attention-aware models showed nearly identical precision-recall performance for the integrated and CIBMTR cohorts (Fig. 1d).

SHapley Additive exPlanations (SHAP) analyses highlighted recurrent features contributing to model performance (Fig. 1e, S4b–e). Across TabPFN models, white blood cell (WBC) count and differentials contributed most significantly to mobilizer prediction, with additional contributions from other cell counts (red blood cells, platelets), age, and sex. These findings are aligned with prior reports that advanced age and lower leukocyte counts predict reduced CD34^+^ cell yields [3–5, 9]. Although SHAP analysis of the 42 variable pre-GCSF dataset identified several chemistry values contributing to model performance, these labs appeared dispensable given the retained model accuracy in their absence. This direct model-to-model comparison of feature importance suggests that donor demographic and CBC indices alone may be used to predict donor mobilization success, irrespective of the evaluation timepoint.

## Discussion

This study evaluates the performance of transformer-based and attention-aware deep learning models for HSPC mobilization outcomes using pre- and post-mobilization laboratory data. We found that (i) TabPFN achieves excellent discrimination with baseline features (AUC = 0.96), (ii) performance was retained with only 14 core demographic and CBC variables (AUC = 0.96), and (iii) post-mobilization data further improves accuracy (AUC = 0.99). Finally, our unified attention-aware model, integrating both timepoints via a lab-type flag and feature-level attention, produces robust discrimination (AUC = 0.89) while offering interpretable context-specific feature weighting. Notably, the attention aware model yielded a slightly lower AUC, reflecting the challenge of integrating heterogeneous pre- and post-mobilization features. These performances exceed those reported for prior regression- and machine-learning-based scores, which achieved more modest discrimination despite using similar classes of predictors [4]. Importantly, influential variables align with those evaluated in prior studies, strengthening confidence that the observed relationships are biologically meaningful [3, 10].

Furthermore, the attention-aware model provides an integrated solution for mixed time-points by re-weighting features according to the lab context flag, providing an advantage in real-world settings where centers collect laboratory data at different stages of donor evaluation and mobilization and wish to apply a single decision tool across time. To test this generalizability, we applied our attention-aware framework to a large, publicly available dataset of over 19,000 healthy donors compiled by the CIBMTR [8]. Despite differences in feature definitions and population composition, the model achieved an accuracy of 79%, demonstrating its applicability across diverse real-world donor populations.

We propose several clinical utilities of these established models. (1) For centers routinely gathering baseline healthy donor CBC data, the pre-mobilization TabPFN prediction model can accurately identify donors who are likely to poorly mobilize, facilitating the selection of alternative donors or preemptive modification of mobilization regimen, such as intensified G-CSF dosing or addition of plerixafor. (2) As novel short-acting mobilizing agents are developed, centers may utilize the high-accuracy post-mobilization TabPFN model to triage the addition of these “just-in-time” pharmacologic tools. For example, in preclinical and clinical studies, the selective CXCR4 antagonist motixafortide (BL-8040, BKT140) rapidly increased PB CD34^+^ cell counts, peaking at 2–4 h after the first dose [11]. Very late antigen 4 (VLA4) inhibitors (BIO5192, BOP) and a CXCR2 agonist (MGTA-145/GROβT) have also demonstrated efficacy as rapid mobilizing strategies [12]. In the future, it is likely that incorporation of donor mobilization prediction strategies coupled with rapid rescue mobilization agents will reduce failed collections, shorten mobilization courses, and curb unnecessary apheresis sessions. (3) Finally, as standards of practice for evaluating donors vary across institutions, the attention-aware model offers clinicians flexibility in predicting mobilization success and offering intervention at several timepoints. Altogether, this study offers a foundation for prospective validation and eventual integration of data-driven decision support into HSPC donor management.

## Supplementary information

Supplemental Material

Table S1

## Data Availability

Data supporting the findings of this study can be obtained from the corresponding author upon reasonable request.

## Code availability

All codes and trained models are available at: https://www.github.com/asif7adil/deepmop.
