# Supplementary material for: Advanced deep learning enables prediction of allogeneic stem cell mobilization success
Source: Bone Marrow Transplant. 2026 Mar 17;61(5):601–4. doi: 10.1038/s41409-026-02811-6 (PMC13152803; doi:10.1038/s41409-026-02811-6)
Supplement: Supplementary file 1 — Supplemental Material [file 41409_2026_2811_MOESM1_ESM.pdf]

Advanced Deep Learning Enables Prediction of Allogeneic Stem Cell Mobilization Success

Asif Adil [1,2], Jingyu Xiang [3], Nicola Piccirillo [4,5], Hillary G. Harris [1], Simona Sica [4,5], John F. DiPersio [3], Stephanie N. Hurwitz [1,2,#]

[1] Department of Pathology and Laboratory Medicine, Indiana University, Indianapolis, IN, USA

[2] Melvin and Bren Simon Comprehensive Cancer Center, Indianapolis, IN, USA

[3] Division of Oncology, Department of Medicine, Washington University School of Medicine, St. Louis, MO, USA

[4] Dipartimento di Scienze di Laboratorio ed Ematologiche, Fondazione Policlinico Universitario "A. Gemelli" IRCCS, Rome, Italy

[5] Sezione di Ematologia, Dipartimento di Scienze Radiologiche ed Ematologiche, Università Cattolica del Sacro Cuore, Rome, Italy

[#] Corresponding author:

Stephanie N. Hurwitz, MD, PhD

Indiana University School of Medicine

Walther Hall, C321C

980 West Walnut St, Indianapolis, IN 46202

sthurw@iu.edu

Supplementary Methods:

Mobilization threshold definition

To establish a binary definition of mobilization quality independent of recipient variables, donor PB $CD34^+$ cell/µL measured on the day of collection was compared to total $CD34^+$ cells/recipient kilogram (kg) weight collected. This analysis was performed using the IU donor subset mobilized with GCSF alone (n = 170). A strong positive correlation between these values was observed, supporting the use of $CD34^+$ cell/µL as a donor-dependent outcome metric, as previously described (1,2). Based on total $CD34^+$ cells/recipient kilogram weight, donors were categorized into "good" ($\geq 5 \times 10^6$), and "poor" ($<5 \times 10^6$). Median yields were calculated for each category of donors to establish the cutoff of ≥40 $CD34^+$ cells/µL to define "good mobilizers," a threshold that is consistent with that used previously (1).

Data integration for the attention aware deep learning model

To generate the unified dataset required for training our self attention–aware deep learning model, donor records from IU, WU, and CU were merged, including 14 variables common to all cohorts: 12 CBC indices, donor age, and biologic sex (**Table S2**). We focused on these variables as they are routinely collected on nearly all donors, and have variably been reported to predict mobilization success. Continuous variables were scaled to the 0–1 interval using min–max normalization. Detailed information on G-CSF formulation and dosing, timing of additional mobilizing agents, and apheresis device or procedural settings was not available in a standardized format across centers and was therefore not included as model input. Two-tailed Student's t-tests were used to compare key features between good vs. poor mobilizer groups. All the results were considered significant if p-value was <0.05.

Feature engineering

To enhance the predictive performance of models trained on datasets with a limited number of input features, we employed several feature engineering strategies, including one-hot encoding (3), feature derivation, and imputation techniques. One-hot encoding was applied to categorical variables such as "Age-group" and "BMI-group" in the CIBMTR donor dataset (4) , allowing the model to interpret group-based differences without imposing ordinal assumptions. Additionally, derived features were constructed to enrich the biological relevance of the input space and to address missing values using clinically valid relationships. For example, neutrophil percentage was calculated from total WBC and absolute neutrophil count when not explicitly available. These computed values allowed for consistent representation across donors and preserved important immunological signals relevant to mobilization biology. All numerical features, including derived variables, were standardized prior to model training to ensure numerical stability and prevent feature scale bias.

Handling class imbalance

Because the datasets exhibited class imbalance (disproportionate numbers of good vs. poor mobilizers), we applied the Synthetic Minority Oversampling Technique (SMOTE) (5) to the training data to balance class frequencies. SMOTE generates synthetic minority-class examples by interpolating between existing minority samples. This oversampling approach was restricted to the

model training phase to avoid information leakage. By achieving an approximately 1:1 class ratio in the training set, we aimed to prevent biased model learning and improve generalization to both mobilization outcomes.

Attention aware deep-learning architecture

A self-attention–aware feed-forward network was implemented in TensorFlow/Keras to classify donors as good or poor mobilizers. The model accepts two inputs: feature vector **(***feat_in***), lab-type flag** (*lab_in*). To allow the network to weight predictors differently according to sampling context, we introduced a *FeatureAttention* layer which concatenates the feature vector with the lab-type flag and passes the joint representation through a *tanh*-activated hidden dense layer (*units* = *n_feat*). A subsequent *soft-max* layer yields an attention vector $\boldsymbol{\alpha}$ of length *n_feat*; element-wise multiplication ($feat \times \alpha$) produces a context-weighted feature set that is forwarded to the classifier and retained for interpretability through trained attention extractor (*att_extractor*) enabling context-specific feature-importance analysis for pre- and post-G-CSF samples.

The downstream classifier comprises four fully connected layers with *leaky-ReLU* or *ReLU* activations, L2 weight regularization ($\lambda = 0.02$) on the first two layers, and batch normalization follows the initial block. Dropout rates, 0.1–0.2 are used sequentially to mitigate overfitting. Finally, a *sigmoid node* outputs the mobilization probability.

In a second model trained on CIBMTR donor dataset (n = 19,207), the architecture was deepened with six layers with decreasing units, each using leaky ReLU activations and followed by dropout (0.1–0.2), L2 regularization ($\lambda = 4.5 \times 10^{-6}$) and batch normalization where appropriate. This hierarchical structure allows the network to capture non-linear relationships among features while maintaining robustness against overfitting.

Both networks were trained using the *Adam optimizer* ($learning\_rate = 1 \times 10^{-4}$) with a binary-cross-entropy loss. Label smoothening was incorporated to reduce overconfidence in predictions. Model training was performed on the balanced training set with early stopping on the validation performance to minimize overfitting. Performance was monitored using ROC-AUC and binary accuracy metrics.

Model evaluation and explainability

For training and testing TabPFN, we used an 80:20 stratified train-test split to maintain class proportions. Model performance was then evaluated on a hold-out test set comprising 20% of the data. Importantly, because TabPFN necessitates minimal hyperparameter optimization, we were able to reserve the entire 20% hold-out set for independent testing. For the attention-aware model, we used an 80:20 train-test split; out of the 20% we carved 80% for validation and another 20% were utilized for testing. Specifically, the dataset was partitioned with two successive stratified random splits (random_state = 42), yielding 80% for model training, 16% for validation, and an untouched 4% hold-out set for final testing; all preprocessing/resampling was fitted on the training set only, with hyperparameters chosen on the validation set and the test set used once for final evaluation.

We assessed several performance metrics on the test set, including the area under the receiver operating characteristic curve (ROC–AUC), overall accuracy, precision, recall, F1-score and Mathews Correlation Coefficient (MCC), defined as:

$$MCC = \frac{TP \ \times TN - FP \ \times FN}{\sqrt{(TP + FP)(TP + FN)(TN + FP)(TN + FN)}} \tag{1}$$

Here, TP, FP, TN, and FN denote true positives, false positives, true negatives, and false negatives, respectively. The MCC is a correlation coefficient ranging from −1 (complete disagreement) through 0 (chance-level) to +1 (perfect prediction).

To interpret how model predictions were made, we employed SHAP (SHapley Additive Explanations) analysis. SHAP assigns each feature an importance value for individual predictions, enabling a game-theoretic understanding of the contribution of each predictor to the model's output (6). The SHAP package (6) was used to compute SHAP values for TabPFN-based models to identify which donor features most strongly influenced the mobilization outcome predictions. These interpretations were based on top 200 donor instances for pre-G-CSF (baseline) data trained model, and all test set instances (n=119) for the post-mobilization trained TabPFN model.

Evaluation of attention mechanism performance

To assess the quality and context sensitivity of the attention mechanism, we employed two complementary metrics: attention entropy and domain-specific attention divergence (**Supplemental Figure 5**). Attention entropy, computed for each sample as

$$H(\alpha) = -\sum_{i=1}^{n} \alpha_i \log(\alpha_i) \tag{2}$$

where $\alpha = [\alpha_1, \alpha_2, \alpha_3, \dots, \alpha_n]$ denotes the attention weights assigned to the n input features for a given sample. This quantifies the sharpness or focus of the attention distribution over input features. Lower entropy values indicate concentrated attention on a smaller subset of predictors, suggesting higher model interpretability and stronger feature selectivity. In contrast, higher entropy implies diffuse attention across many features, reflecting model uncertainty or weak discriminative preference. Moderate entropy values, as observed in our study, suggest a balance in which the model selectively attends to informative features while maintaining flexibility, supporting both interpretability and generalizability.

To assess domain adaptability, we computed the Jensen–Shannon (JS) divergence between the mean attention vectors for pre- and post-G-CSF samples. A higher JS divergence reflects more distinct attention patterns across lab types, confirming the network's ability to modulate feature importance based on sampling context. Feature-wise differences in attention were also analyzed to identify predictors differentially emphasized between domains.

Supplementary Figure 1:

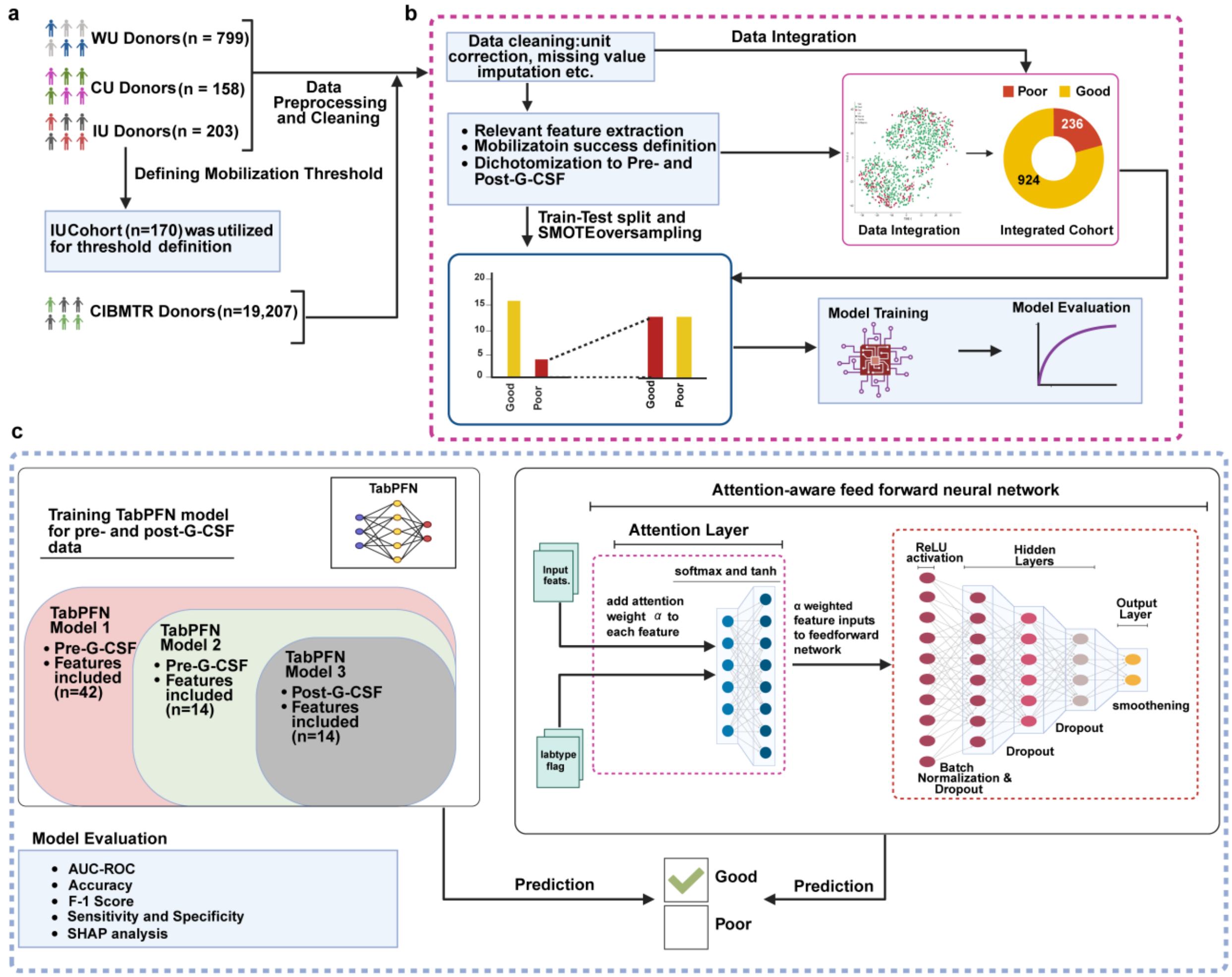

Figure 1. Schematic overview of the analytical pipeline. a) Data acquisition and preprocessing. Peripheral blood counts and demographics were collated from three independent centers (Washington University, WU; Catholic University, CU; Indiana University, IU and CIBMTR, Center for International Blood and Marrow Transplant Research). An empirical mobilization threshold was first defined in an IU subset (n = 170). **b)** All cohorts then underwent unit harmonization, missing-value imputation, and SMOTE-based oversampling to correct class imbalance before an 80:20 stratified train/test split. After batch correction, the three datasets were merged into an integrated cohort (good mobilizers = 924, poor mobilizers = 236) for exploratory analyses. **c)** Model development and evaluation. Left: Three TabPFN models were trained: (1) full pre-G-CSF feature set (42 variables); (2) reduced pre-G-CSF panel (14 variables); and (3) post-G-CSF panel (14 variables). Right: An attention-aware feed-forward neural network leveraging the "lab-type" domain indicator was trained in parallel. Each model outputs a binary prediction (good vs poor) that is assessed by precision–recall curves, conventional performance metrics, and XAI-based feature attribution.

Supplementary Figure 2

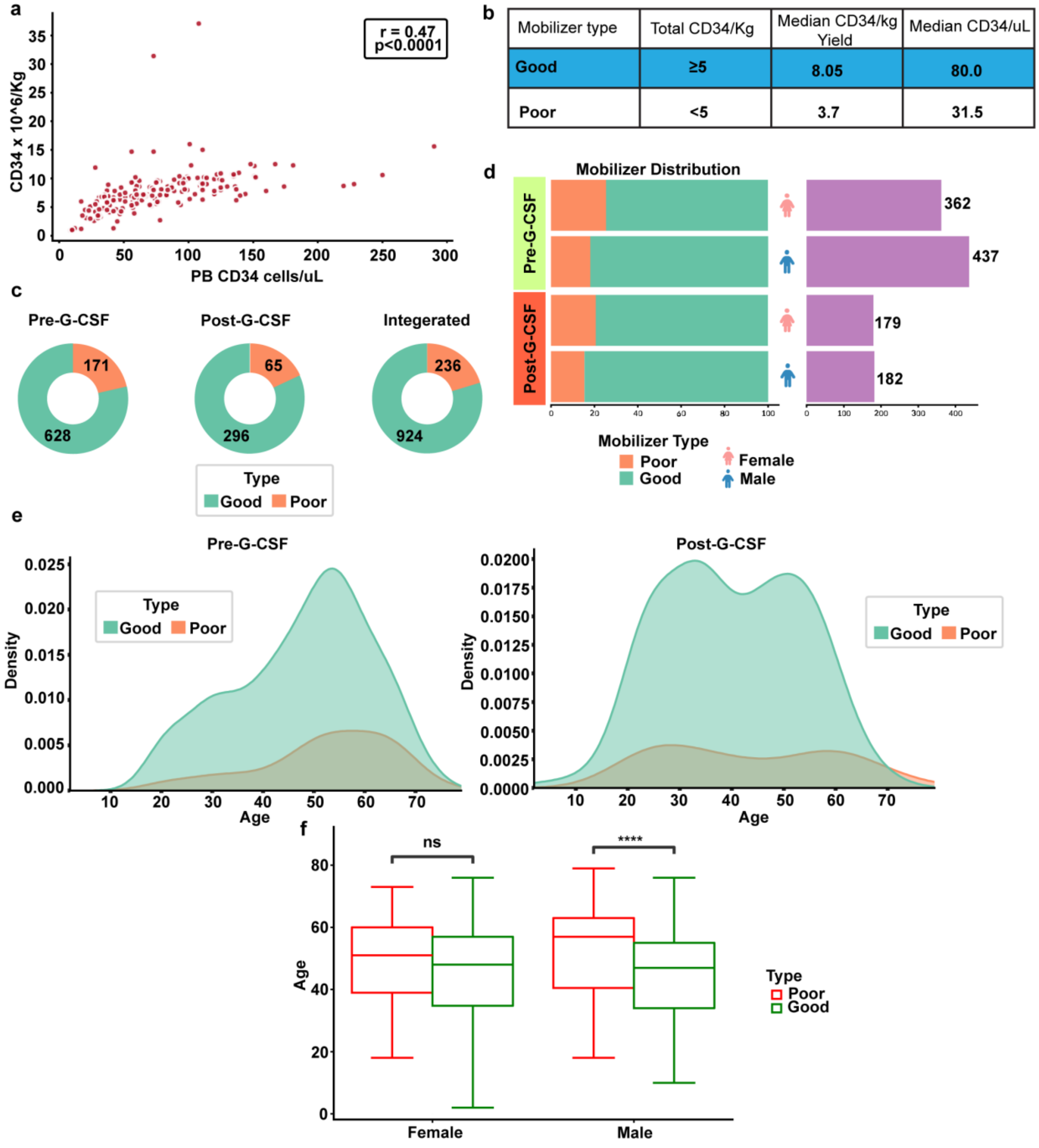

Figure 2. Characterization of donor mobilization efficiency across G-CSF timepoints and associated demographic patterns. a) Correlation of IU GCSF-mobilized donor peripheral blood $CD34^+$ counts (day of collection) with total $CD34^+$ cell/recipient kg yield (r = 0.47, p < 0.0001). **b)** Table showing the median values of Day 1 PB $CD34^+$ count and yield across mobilizer categories. **c)** Frequency of good and poor mobilizers in the pre- and post-G-CSF cohorts, based on a 40 $CD34^+$ cell/µL threshold. **d)** Distribution of good and poor mobilizers across cohorts and by sex. **e)** Kernel-density plots of donor age across mobilizer type in the pre- and post-G-CSF cohorts. **f)** Box-and-whisker plots comparing age and sex as mobilization factors. ****, $p < 0.0001$; ns, not significant.

Supplementary Figure S3

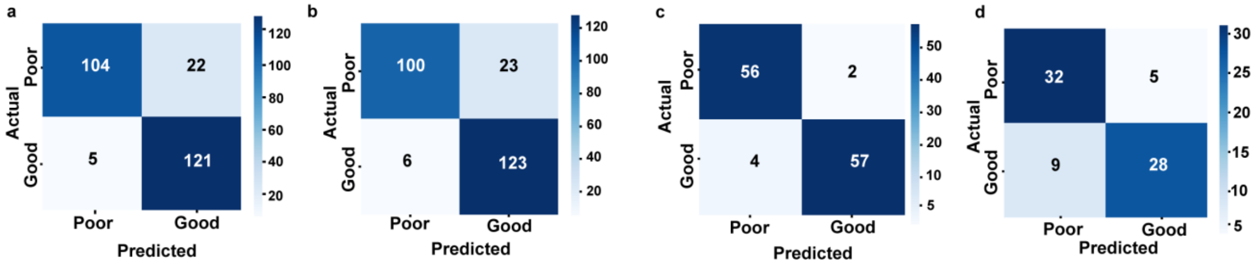

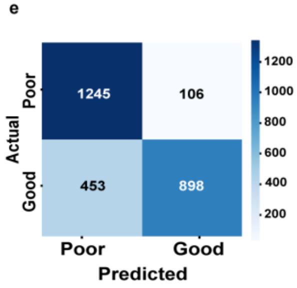

f

Model Type	Accuracy (%)	AUC (%)	Precision (%)	Recall (%)	F1-Score (%)	MCC
Pre-G TabPFN (42 var)	89	96	85	96	90	0.71
Pre-G TabPFN (14 var)	91	96	89	94	91	0.83
Post-G TabPFN (14 var)	94	99	96	93	95	0.89
Attention Aware (IC)	81	89	85	76	80	0.63
Attention Aware (CIBMTR)	79	88	89	66	76	0.60

Figure 3. Performance of machine learning algorithms on pre- and post-GCSF data. a) Confusion matrices for TabPFN models trained on **(a)** the 42 variable pre-G-CSF donor dataset, **(b)** the 14 variable pre-G-CSF dataset, and **(c)** the 14 variable post-G-CSF dataset. Confusion matrices for attention-aware MLP evaluated in the **(d)** integrated multi-institutional and **(e)** the external CIBMTR cohorts. **(f)** Summary of performance metrics across models. Pre-G, Pre-G-CSF; Post-G, Post-G-CSF; IC, Integrated Cohort.

Supplementary Figure 4

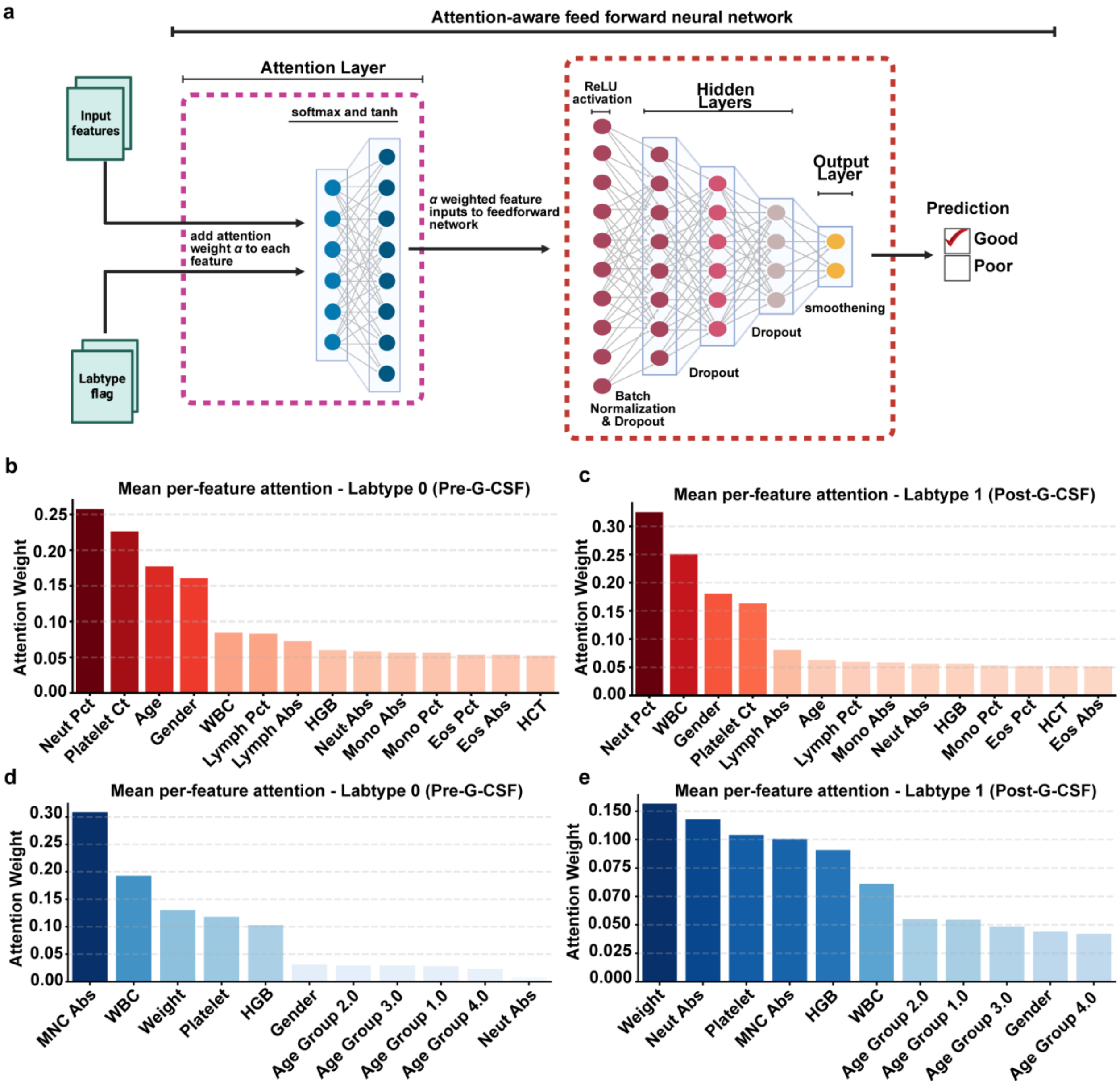

Figure 4. Feature-wise attention profiles across lab contexts. a) Attention-aware feed-forward neural network for donor mobilization prediction. Clinical and complete blood count variables, together with a binary pre-/post-G-CSF flag (Labtype), enter an attention gate that assigns a normalized weight (α) to each feature via a soft-max + tanh mechanism. The re-weighted feature vector is passed to a multi-layer perceptron that incorporates ReLU activation, batch normalization, and dropout regularization. A final sigmoid node outputs the probability of successful ("good") versus inadequate ("poor") $CD34^+$ cell mobilization, while the learned α weights are retained for downstream interpretation of driver features. Mean self-attention weights for samples collected **b)** before GCSF (labtype 0) or **c)** after GCSF administration (labtype 1), in integrated cohort. **d)** Mean per-feature attention weights for (labtype 0) pre-G-CSF, and **(e)** labtype 1 (post-G-CSF) of model trained on CIBMTR Cohort.

Supplementary Figure 5:

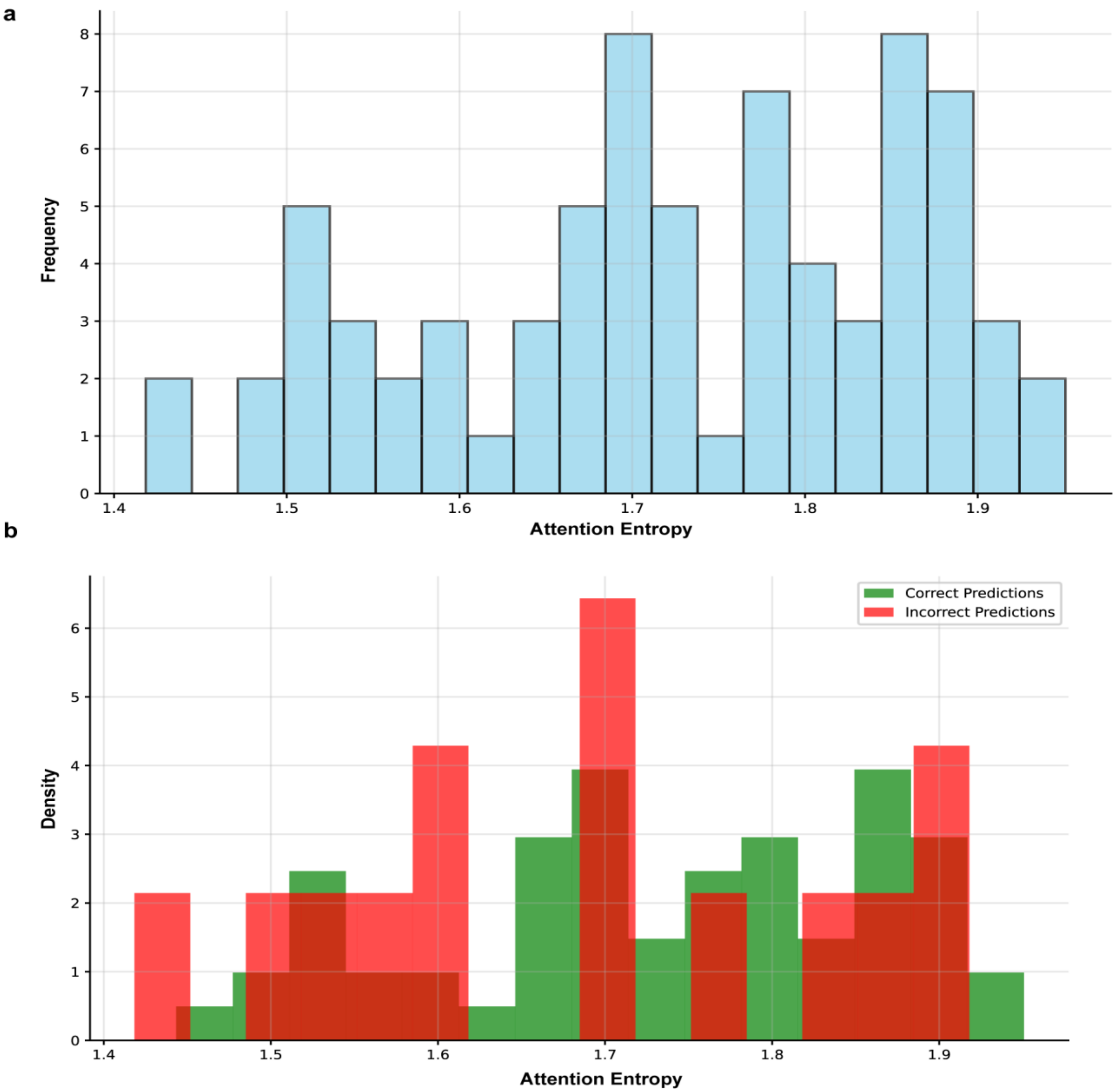

Figure 5. Distributions of attention entropy across prediction outcomes on integrated cohort. (a) Histogram showing the overall distribution of attention entropy values across all samples. The mean attention entropy was 1.73 (natural log scale), suggesting that the model neither distributed its attention uniformly across all features nor overly concentrated it on a single input. **(b)** Density histogram stratified by prediction accuracy, highlighting the attention entropy distribution for correctly classified (green) versus incorrectly classified (red) samples. Entropy distributions were comparable between correct and incorrect predictions (1.73 vs. 1.69). Negligible correlation between attention entropy and prediction confidence (Pearson's r=0.019, p=0.87) supported an independent learned mechanism. Finally, Jensen–Shannon divergence between attention distributions in pre- and post-G-CSF samples was 0.274, confirming that the

attention mechanism adapted meaningfully to the lab context while maintaining shared predictive structure.

References:

1. Xiang J, Shi M, Fiala MA, Gao F, Rettig MP, Uy GL, et al. Machine learning–based scoring models to predict hematopoietic stem cell mobilization in allogeneic donors. Blood Adv. 2022 Mar 25;6(7):1991–2000.

2. To LB, Levesque JP, Herbert KE. How I treat patients who mobilize hematopoietic stem cells poorly. Blood. 2011 Oct 27;118(17):4530–40.

3. Poslavskaya E, Korolev A. Encoding categorical data: Is there yet anything "hotter" than one-hot encoding?. arXiv; 2023. http://arxiv.org/abs/2312.16930. Accessed 26 Jan 2026

4. Hsu JW, Shaw BE, Kim S, Logan BR, Sees JA, Confer DL, et al. Collection of Peripheral Blood Progenitor Cells in 1 Day Is Associated with Decreased Donor Toxicity Compared to 2 Days in Unrelated Donors. Biol Blood Marrow Transplant. 2020 Jun 1;26(6):1210–7.

5. Chawla NV, Bowyer KW, Hall LO, Kegelmeyer WP. SMOTE: Synthetic Minority Over-sampling Technique. J Artif Intell Res. 2002 Jun 1;16:321–57.

6. Lundberg SM, Lee SI. A Unified Approach to Interpreting Model Predictions. In: Advances in Neural Information Processing Systems. Curran Associates, Inc. 2017. https://papers.nips.cc/paper_files/paper/2017/hash/8a20a8621978632d76c43dfd28b67767-Abstract.html. Accessed 26 Jan 2026
